# Human Adipose Tissue Conditioned Media from Lean Subjects Is Protective against H_2_O_2_ Induced Neurotoxicity in Human SH-SY5Y Neuronal Cells

**DOI:** 10.3390/ijms16011221

**Published:** 2015-01-06

**Authors:** Zhongxiao Wan, Dorrian Mah, Svetlana Simtchouk, Andreas Kluftinger, Jonathan P. Little

**Affiliations:** 1 School of Health and Exercise Sciences, University of British Columbia Okanagan, Kelowna, BC V1V 1V7, Canada; E-Mails: zhongxiao.wan@ubc.ca (Z.W.); dorrian_mah@hotmail.com (D.M.); simtchouk@yahoo.ca (S.S.); 2 Department of Surgery, University of British Columbia; General, Laparoscopic, Endocrine and Gastric Band Surgery, 203-3040 Tutt St., Kelowna, BC V1Y 2H5, Canada; E-Mail: kluftin@shaw.ca

**Keywords:** obesity, adipokine, Alzheimer’s disease, oxidative stress

## Abstract

Adipose tissue secretes numerous hormone-like factors, which are known as adipokines. Adipokine receptors have been identified in the central nervous system but the potential role of adipokine signaling in neuroprotection is unclear. The aim of this study is to determine (1) Whether adipokines secreted from cultured adipose tissue of lean humans is protective against oxidative stress-induced neurotoxicity in human SH-SY5Y neuronal cells; and (2) To explore potential signaling pathways involved in these processes. Adipose tissue conditioned media (ATCM) from healthy lean subjects completely prevented H_2_O_2_ induced neurotoxicity, while this effect is lost after heating ATCM. ATCM activated the phosphorylation of ERK1/2, JNK and Akt at serine 308 in SH-SY5Y cells. PD98059 (25 µM), SP600125 (5 µM) and LY29400 (20 µM) partially blocked the protective effects of ATCM against H_2_O_2_ induced neurotoxicity. Findings demonstrate that heat-sensitive factors secreted from human adipose tissue of lean subjects are protective against H_2_O_2_ induced neurotoxicity and ERK1/2, JNK, and PI3K signaling pathways are involved in these processes. In conclusion, this study demonstrates preliminary but encouraging data to further support that adipose tissue secreted factors from lean human subjects might possess neuroprotective properties and unravel the specific roles of ERK1/2, JNK and PI3K in these processes.

## 1. Introduction

Alzheimer’s disease (AD) is the leading cause of dementia for people aged over 60. The global prevalence of dementia in this age group is predicted to double every 20 years, affecting more than 80 million people worldwide by 2040 [1]. As yet, neither a satisfying therapy nor a preventative cure is available for AD. This is largely because our understanding of the complex biology of AD is incomplete, highlighting the importance of exploring novel mechanisms underlying AD.

Although traditionally regarded as an inert storage depot for excess lipids, it is now well-accepted that adipose tissue is an active endocrine organ that secretes a host of hormone-like substances, termed adipokines [2], which can crosstalk with and affect the function of distant organs/tissues. Adipokine levels from individuals with obesity are altered when compared to lean counterparts with a general reduction in adiponectin and increase in leptin secretion [3]. Multiple adipokine receptors such as adiponectin receptor [4], leptin receptor [5] and resistin receptor [6] have been reported in the central nervous system (CNS). Accumulating evidence suggests that adipokines may play a role in the pathogenesis or prevention of AD. For example, Chan *et al.* [7] reported that adiponectin is protective against amyloid beta (Aβ) induced neurotoxicity in Sw-APP transfected SH-SY5Y cells under oxidative stress conditions. Leptin can ameliorate both Aβ and tau-related pathological pathways in SH-SY5Y and NTERA-2 cells [8]. Benomar *et al.* [6] reported that resistin negatively affects hypothalamic insulin signaling in SH-SY5Y human neuronal cells *in vitro*. Although exciting, most of the current evidence about the connection between adipokines and AD have primarily focused on a single adipokine under supraphysiological levels. Whether secreted factors from adipose tissue holistically are involved in the pathogenesis or prevention of AD remains unknown.

The pathogenesis of AD is multifactorial and complex, and oxidative stress may represent a key factor integrating the divergent nature of multiple pathogenic mechanisms of AD [9]. Therefore, studying strategies that affect neuronal response to oxidative insults could serve as important avenues to evaluate potential preventative or therapeutic agents for AD development.

The PI3K/Akt pathway is a central signal transduction pathway involved in cell growth, survival and metabolism [10]. Many research groups have demonstrated that PI3-K activity is responsible for as much as 80% of neurotrophin regulated cell survival, indicating that PI3-K is a critical survival-promoting protein for neurons [11]. Mitogen-activated protein kinases (MAPKs) including c-Jun *N*-terminal kinases (JNK), extracellular-signal-regulated kinases1/2 (ERK1/2), and p38 MAPK are serine-threonine kinases that mediate intracellular signaling associated with a wide range of cellular activities, including cell proliferation, differentiation, survival, death and transformation. The influence of adipokines on PI3K and MAPK signalling in neurons has not been adequately studied. The aim of this study is to determine (1) Whether adipose tissue conditioned media (ATCM) from lean subjects are protective against oxidative stress-induced neurotoxicity in human SH-SY5Y neuronal cells; and (2) To explore potential signaling pathways involved in these processes. We hypothesized that ATCM from lean subjects would be protective against hydrogen peroxide (H_2_O_2_)-induced neurotoxicity in neuronal cells and that PI3K, p38MAPK, ERK1/2 and JNK signalling would be involved in the protective effects.

## 2. Results and Discussion

### 2.1. Results

#### 2.1.1. Heat Sensitive Factors Secreted from Human Visceral Adipose Tissue Protect against H_2_O_2_ Induced Toxicity in SH-SY5Y Neuronal Cells

**Figure 1:**
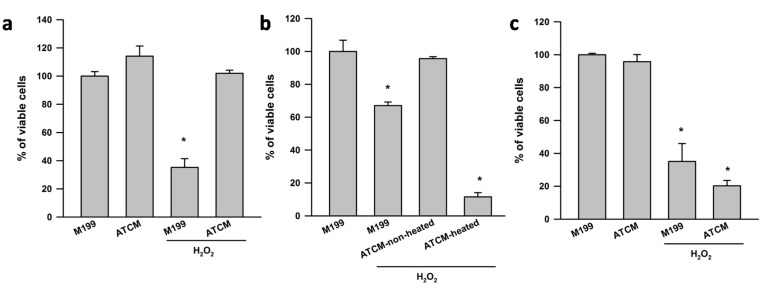
Heat-sensitive factors secreted from human preperitoneal adipose tissue protect against H_2_O_2_ induced toxicity in SH-SY5Y neuronal cells. Adipose tissue conditioned media (ATCM) from lean subjects was applied to SH-SY5Y neuronal cells for 24 h in the presence of 800 µM H_2_O_2_. Control cell cultures were treated with M199 media. (**a**) ATCM had no direct effects on SH-SY5Y neuronal cell viability assessed by the MTT assay, but lean ATCM completely reversed H_2_O_2_ induced neurotoxicity; (**b**) The neuroprotective effect of lean ATCM was abolished when ATCM was heated at 95 °C for 10 min and (**c**) ATCM from subcutaneous fat of obese subjects demonstrated no effect on H_2_O_2_ induced neurotoxicity. Data (mean + S.E.M.) are expressed as % viable cells, where 100% values were obtained from SH-SY5Y cells incubated with fresh M199 only. All cell culture experiments were repeated on two separate passages of cells with ATCM from three different subjects (*n* = 6). * *p* < 0.05 compared to M199 control.

Figure 1a shows the viability of SH-SY5Y cells that has been exposed to ATCM challenged with or without H_2_O_2_. The MTT assay revealed that H_2_O_2_ reduced cell viability to ~35% of control wells treated with fresh media only. ATCM completely prevented H_2_O_2_ induced neurotoxicity. ATCM that had been heated to 95 °C for 10 min showed no protective effects against H_2_O_2_ induced neurotoxicity, suggesting that heat-sensitive factors secreted from human visceral adipose tissue protect against H_2_O_2_ induced toxicity in SH-SY5Y neuronal cells. (Figure 1b). It should be noted that M199 heated condition exerted similar effects as M199 unheated (data not shown). ATCM from the subcutaneous fat depot of obese subjects has no effect on H_2_O_2_ induced neurotoxicity (Figure 1c). The adiponectin (lean: 724 ± 337 *vs.* obese: 458 ± 82 pg/mL/g tissue; *p* > 0.05) and leptin (lean: 298 ± 54 *vs.* obese: 323 ± 148 pg/mL/g tissue, *p* > 0.05) concentration in ATCM from lean and obese subjects were not significantly different.

#### 2.1.2. ERK1/2, JNK and PI3K Are Involved in the Protective Effects of ATCM against H_2_O_2_ Induced Neurotoxicity

**Figure 2:**
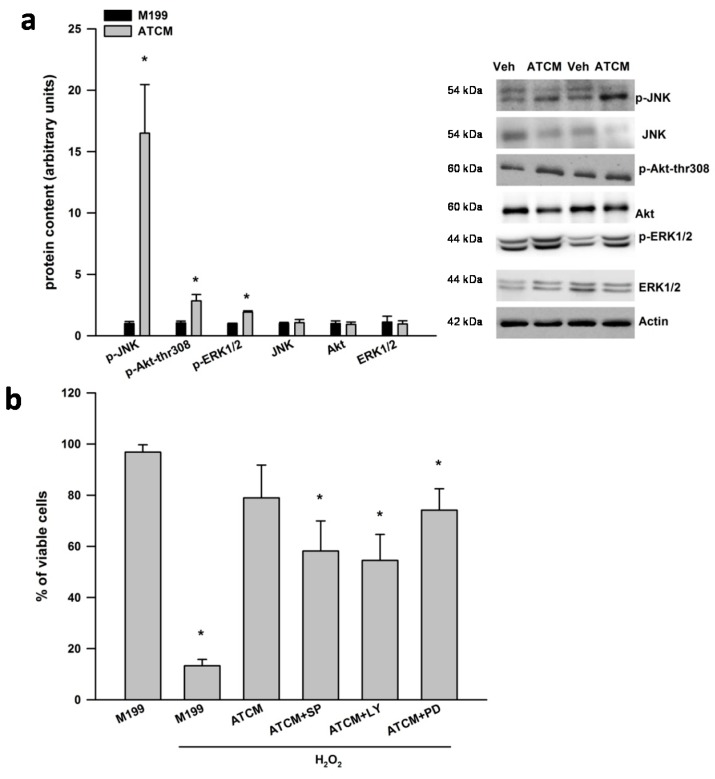
ERK1/2, JNK and PI3K are involved in the protective effects of lean ATCM against H_2_O_2_ induced neurotoxicity. (**a**) Adipose tissue conditioned media (ATCM) from lean subjects was applied to SH-SY5Y neuronal cells for 1 h and the phosphorylation of JNK, Akt at threonine 308, and ERK1/2 was measured by western blotting. Phosphorylated signaling proteins are expressed relative to respective total protein and the ATCM condition was compared to vehicle (veh) treated well and (**b**) Adipose tissue conditioned media (ATCM) from lean subjects was applied to SH-SY5Y neuronal cells for 24 h in the presence of 800 µM H_2_O_2_. Chemical inhibition of JNK with SP600125 (5 µM), PI3-K with LY29400 (20 µM) and ERK with PD98059 (25 µM) partially inhibited the neuroprotective effects of ATCM in SH-SY5Y cells under oxidative stress conditions. Representative western blots are shown to the right of the quantified data in panel A. All cell culture experiments were repeated on two separate passages of cells with ATCM from three different subjects (*n* = 6). * *p* < 0.05 compared to M199 control.

As shown in Figure 2a, lean ATCM treatment for 1 h activated the phosphorylation of ERK1/2, JNK and Akt at serine 308 in SH-SY5Y cells. There were no differences in the total protein content of ERK1/2, JNK and Akt. PD98059, SP600125, LY294002 partially blocked the protective effects of ATCM against H_2_O_2_ induced neurotoxicity (Figure 2b). Overall, these data suggest that signaling pathways via ERK1/2, JNK and PI3K are partially involved in the protective effects of ATCM against H_2_O_2_ induced neurotoxicity.

### 2.2. Discussion

Our current study demonstrates that heat-sensitive factors secreted from human adipose tissue of lean subjects are protective against H_2_O_2_ induced neurotoxicity and ERK1/2, JNK, and PI3K signaling pathways are potentially involved in these processes. These findings in our model system of human adipose-brain crosstalk indicate the potential for adipokines to have neuroprotective functions by activating certain signalling pathways in neuronal cells.

Oxidative stress plays a significant role in the pathogenesis of several neurodegenerative diseases including AD [9]. H_2_O_2_ induced neurotoxicity in SH-SY5Y cells has been widely used as a suitable cellular model for investigating novel strategies that affect neuronal responses to oxidative stress injury [12,13]. Our current study demonstrates that heat-sensitive factors secreted from adipose tissue of lean human subjects are protective against H_2_O_2_ induced neurotoxicity in SH-SY5Y cells, which extends findings from other research groups. For example, Lu *et al.* [14] reported that adipose derived mesenchymal stem cells (MSCs) are capable of protecting PC12 cells from glutamate excitotoxicity via activation of the PI3K/Akt pathway. Multiple factors secreted from adipose tissue could be involved in protecting neuronal cells from toxic insults and our data and this previous study [15] indicate that activation of the PI3K pathway may be involved. Rehman *et al.* [16] has reported that MSCs from human subcutaneous fat secrete angiogenic and antiapoptotic factors including vascular endothelial growth factor (VEGF), hepatocyte growth factor (HGF), transforming growth factor β (TGF-β).Taken together, our findings support the notion that a cocktail of heat-sensitive peptides/proteins secreted from human adipose tissue can protect SH-SY5Y neuronal cells from oxidative stress-induced cell death.

Adipose tissue has been widely accepted as an active endocrine organ that secretes a host of adipokines [2]. Accumulating evidence supports that adipokines might affect brain function. For example, Chan *et al.* [7] reported that high concentrations of adiponectin (10 µg/mL) were protective against amyloid beta induced neurotoxicity in Sw-APP transfected SH-SY5Y cells under oxidative stress conditions. Leptin also may possess neuroprotective abilities in AD, such as protection against hippocampal synaptic disruption and neuronal cell death [17] and inhibition of amyloid beta accumulation [18]. Apelin is another adipokine that can impede neuronal apoptosis in mouse cortical neurons via suppression of ROS generation [19]. Adipose tissue also secretes insulin-like growth factors 1 (IGF-1) [15], which has many functions including promotion of cell survival and inhibition of apoptosis [20,21]. Presumably, all these factors working in combination can interact with each other to exert protective effects against oxidative stress in SH-SY5Y cells. Unfortunately, due to the limitation in human adipose tissue samples, we were unable to perform the time-consuming and tedious experiments that would be required to isolate the individual contribution from each of these potential factors. In addition, we were only able to collect ATCM from subcutaneous fat of obese subjects. Although ATCM from subcutaneous fat of obese subjects has no protective effect against H_2_O_2_ induced neurotoxicity in SH-SY5Y cells, it remains to be conclusively determined if this is owing to depot-specific effect or because dysfunctional adipokine secretion under obese conditions. We noted a tendency for reduced adiponectin and increased leptin secretion from obese subjects, but this did not reach statistical significance. Future research is therefore required (1) to help identify the combination of factors secreted from adipose tissue that possess neuroprotective properties; (2) to explore whether ATCM from different adipose depots and under different metabolic characteristics (such as lean *vs.* obese) might exert effects differently on H_2_O_2_ induced neurotoxicity; and (3) whether the concentrations of adipokines in the brain *in vivo* would elicit the same effects as our model system.

The PI3K/Akt pathway is a central signal transduction pathways involved in cell growth, survival and metabolism [10]. Many research groups have demonstrated that PI3-K activity is responsible for as much as 80% of neurotrophin regulated cell survival, indicating that PI3-K is the major survival-promoting protein for neurons [11]. Similarly, JNK and ERK1/2 activation have a pro-survival effect in multiple cell types [22,23,24]. Our current findings suggest that PI3K, ERK1/2 and JNK signaling pathways are also involved in the protective effects of ATCM against oxidative stress in SH-SY5Y cells.

Our model system used intact human adipose tissue organ cultures to study the potential impact of adipokines on neuronal cells. The benefits of this approach are that the *in vivo* composition of adipose tissue is maintained and the culture conditions allow for intercellular communication amongst the various different cell types within adipose tissue [25]. However, a limitation with this technique is that it cannot determine which cells (e.g., adipocytes, MSCs, immune cells, endothelial cells *etc.*) are responsible for secretion of neuroprotective factors. Future research is required to determine which cell(s) are involved in adipose-brain crosstalk via adipokines.

## 3. Experimental Section

### 3.1. Materials

The following reagents were obtained from ThermoFisher Scientific (Ottawa, ON, Canada): bovine serum albumin (BSA), Dulbecco’s modified Eagle medium nutrient mixture F-12 Ham (DMEM-F12) and Trypsin/EDTA solution. PD98059 (cat#10006726), SP600125 (cat#10010466) and LY294002 (cat#70920) were obtained from Cayman chemicals (Burlington, ON, Canada). Antibodies against phosphorylation-ERK1/2 (p-ERK1/2) (cat#4370), p-JNK (cat#4668), p-Akt serine473 (cat#4060), ERK1/2 (cat#4695), JNK (cat#9258), Akt (cat#4691), and beta-actin (cat#4970), protease/phosphatase inhibitor cocktail (100X) (cat#5872S), phenylmethylsulfonyl fluoride (PMSF; cat#8553), SignalFire ECL Reagent (cat#6883S) and cell lysis buffer (cat#9803) were from Cell Signaling (Danvers, MA, USA). Human enzyme-linked immunosorbent assay (ELISA) Duoset kits for adiponectin (cat#DY1065) and leptin (cat#DY398) were from R&D systems (Minneapolis, MN, USA). Western blotting stripping buffer (cat#46430) was from Millipore (Etobicoke, ON, Canada). Reagents, molecular weight markers and nitrocellulose membranes for SDS-PAGE were obtained from Bio-Rad (Mississauga, ON, Canada). All other chemicals were purchased from Sigma (Oakville, ON, Canada).

### 3.2. Human Adipose Tissue Conditioned Media (ATCM) Collection

Preperitoneal adipose tissue samples were obtained from eight lean female subjects (age 52 ± 6 year, body mass index (BMI) 24.1 ± 1.1 kg/m^2^) undergoing abdominal surgeries. Abdominal subcutaneous adipose tissue samples were obtained from three obese female subjects (age 47 ± 3 year, BMI 30.3 ± 2.0 kg/m^2^). Adipose tissue was transported from the surgical suite in sterile phosphate buffered saline (PBS) and processed within one hour. Adipose tissue organ cultures were prepared according to Wan *et al.* [26] with slight modification. Briefly, 500 mg of adipose tissue was minced into ~5–10 mg pieces and placed into culture dishes containing 15 mL of M199 supplemented with 1% antibiotic/antimycotic, 50 μIU/mL insulin and 2.5 nM dexamethasone. The cultures were placed in a cell incubator at 37 °C to equilibrate for 24 h. On the next morning, media was replaced with fresh M199, in the absence of insulin and dexamethasone. After another 24 h, ATCM were collected and stored at −80 °C for subsequent analyses and treatment of neuronal cells. The present study was conducted according to the guideline laid down in the Declaration of Helsinki and all procedures were approved by University of British Columbia Clinical Research Ethics Board (H12-02330). Written informed consent for obtaining surgical samples was obtained from all subjects.

### 3.3. Cell Culture

The human neuroblastoma SH-SY5Y cells were a kind gift from A. Klegeris (Department of Biology, University of British Columbia Okanagan). Cells were grown in DMEM-F12 supplemented with 10% FBS. Adherent SH-SY5Y cells were detached from the bottom of the flask by incubating cells in 2 mL of 0.05% trypsin/EDTA solution for 5–10 min. The flask was then washed with 10 mL of F10 medium and cell suspension was harvested for experiments. SH-SY5Y cells were counted, centrifuged at 450× *g* for 7 min, and re-suspended in F5 media to a final concentration of 0.2 million/mL for use in experiments. A volume of 0.4 mL/well of re-suspended SH-SY5Y cells were plated in sterile 24-well plates and incubated for 24 h to allow cells to adhere to the plate prior to treatment. For adipose-brain crosstalk experiments, media from SH-SY5Y neuronal cells were aspirated following 24 h incubation and replaced with 400 µL ATCM for 2 h. SH-SY5Y cells were then challenged with H_2_O_2_ (800 µM) and 24 h later an MTT cell viability assay was performed. To help determine if the effects of ATCM were due to heat sensitive factors, parallel experiments were performed where ATCM was heated for 10 min at 95 °C in order to denature proteins/peptides. For experiments with pharmacological inhibitors, the same procedures were followed except that PD98059 (25 µM), SP600125 (5 µM) and LY29400 (20 µM) were administered to SH-SY5Y cells for 1 h following the 2 h pretreatment with ATCM and H_2_O_2_ challenge. For the activation of signaling pathways by ATCM experiment, a volume of 3 mL/well of re-suspended SH-SY5Y cells was plated in sterile 6-well plates and incubated for 24 h to allow cells to adhere to the plate prior to treatment. SH-SY5Y supernatants were aspirated following 24 h incubation and replaced with 3 mL ATCM for 1 h. Thereafter, the cells were washed twice with cold PBS and lysed on ice with cell lysis buffer containing protease and phosphatase inhibitors. All above experiments were repeated at least two times with ATCM from three different subjects *per* time (*i.e*., ATCM from three subjects were applied to two different passages/flasks of cells).

### 3.4. Cell Viability Assay: Reduction of Formazandye (MTT)

The MTT assay was performed as described by Klegeris *et al.* [27]. Briefly, MTT was added to the SH-SY5Y cell cultures to reach a final concentration of 1mg/mL. Following a 2 h incubation at 37 °C, the dark crystals that formed were dissolved by adding an equal volume of SDS/DMF extraction buffer (20% sodium dodecyl sulphate, 50% *N*,*N*-dimethyl formide, pH 4.7). Subsequently, plates were placed overnight at 37 °C and optical densities (OD) were measured by transferring 100 µL aliquots to 96-well plates at 570 nM. The viable cell values in percent were calculated as follows. Levels in wells exposed to ATCM in the presence or absence of H_2_O_2_ were compared with levels in wells exposed only to M199. The latter values were set at 100%. The lower level of activity, *i.e.*, the value for 0% cell survival, was determined by complete lysing of cells with 1% Triton X-100 prior to addition of the MTT solution. The MTT assay demonstrates linearity in the range of 10^4^ and 10^6^ SH-SY5Y cells.

### 3.5. Western Blot Analysis

SH-SY5Y total cell lysates were prepared in cell lysis buffer supplemented with Protease/Phosphatase Inhibitor Cocktail and PMSF. Protein concentrations were determined using a BCA assay reagent kit. Changes in the protein expression of phosphorylation and total ERK, JNK and Akt were determined by Western blotting as described previously by Wan *et al.* [28]. Briefly, equal amounts of protein (*i.e.*, 20 μg/well) were separated on 10% gels. Proteins were wet transferred to nitrocellulose membranes at 100 volts and subsequently blocked in tris buffered saline/0.1% tween 20 (TBST) supplemented with 5% non-fat dry milk for 1 h at room temperature with gentle agitation. Membranes were incubated in TBST/5% non-fat dry milk supplemented with appropriate primary antibodies (the dilution condition for primary antibodies was 1:1000, *i.e.*, 10 μL primary antibodies diluted in 10 mL 5% BSA) overnight at 4 °C with gentle agitation. The following morning, membranes were briefly washed in TBST and then incubated in TBST/1% non-fat dry milk supplemented with HRP conjugated secondary antibodies for 1 h at room temperature. Signals were detected using enhanced chemiluminesence and were subsequently quantified by densitometry by Gene Tool according to the manufacturer’s instructions (SynGene, ChemiGenius2, PerkinElmer). In regards to protein at the similar molecular weight (*i.e.*, p-ERK1/2 and beta-actin), we derived p-ERK1/2 first, the membranes were then stripped with stripping buffer according to the manufacturer’s instructions, re-blocked, and re-probed with beta-actin antibody. All phosphorylated proteins were expressed relative to their respective total protein. Beta actin was used as an internal loading control for quantification.

### 3.6. Adiponectin and Leptin Measurement

The concentrations of adiponectin and leptin were measured by ELISA according to the manufacturer’s instruction. Adiponectin and leptin concentrations were corrected for tissue weight and reported as pg/mL per g tissue. The coefficient of variation for these assays in our laboratory based on duplicates is <10%.

### 3.7. Statistical Analysis

All data are presented as means ± SEM. Comparisons between M199 control and experimental treatment conditions were analyzed using a one-way ANOVA followed by a post hoc comparison using Fishers least significant difference (LSD) test. Western blot data was analyzed by independent *t*-test to compare M199 control to ATCM. The level of statistical significance was set at *p* < 0.05.

## 4. Conclusions

In conclusion, the present study demonstrates preliminary but encouraging data to further support that adipose tissue secreted factors from lean human subjects might possess neuroprotective properties and unravel the specific roles of ERK1/2, JNK and PI3K in these processes.
